# Supplementary material for: Targeting Diabetic Retinopathy with Human iPSC-Derived Vascular Reparative Cells in a Type 2 Diabetes Model
Source: Cells. 2025 Aug 30;14(17):1352. doi: 10.3390/cells14171352 (PMC12428381; doi:10.3390/cells14171352)
Supplement: Supplementary file 1 [file cells-14-01352-s001.zip › cells-3802008-re-supplementary.pptx]

## Slide 1
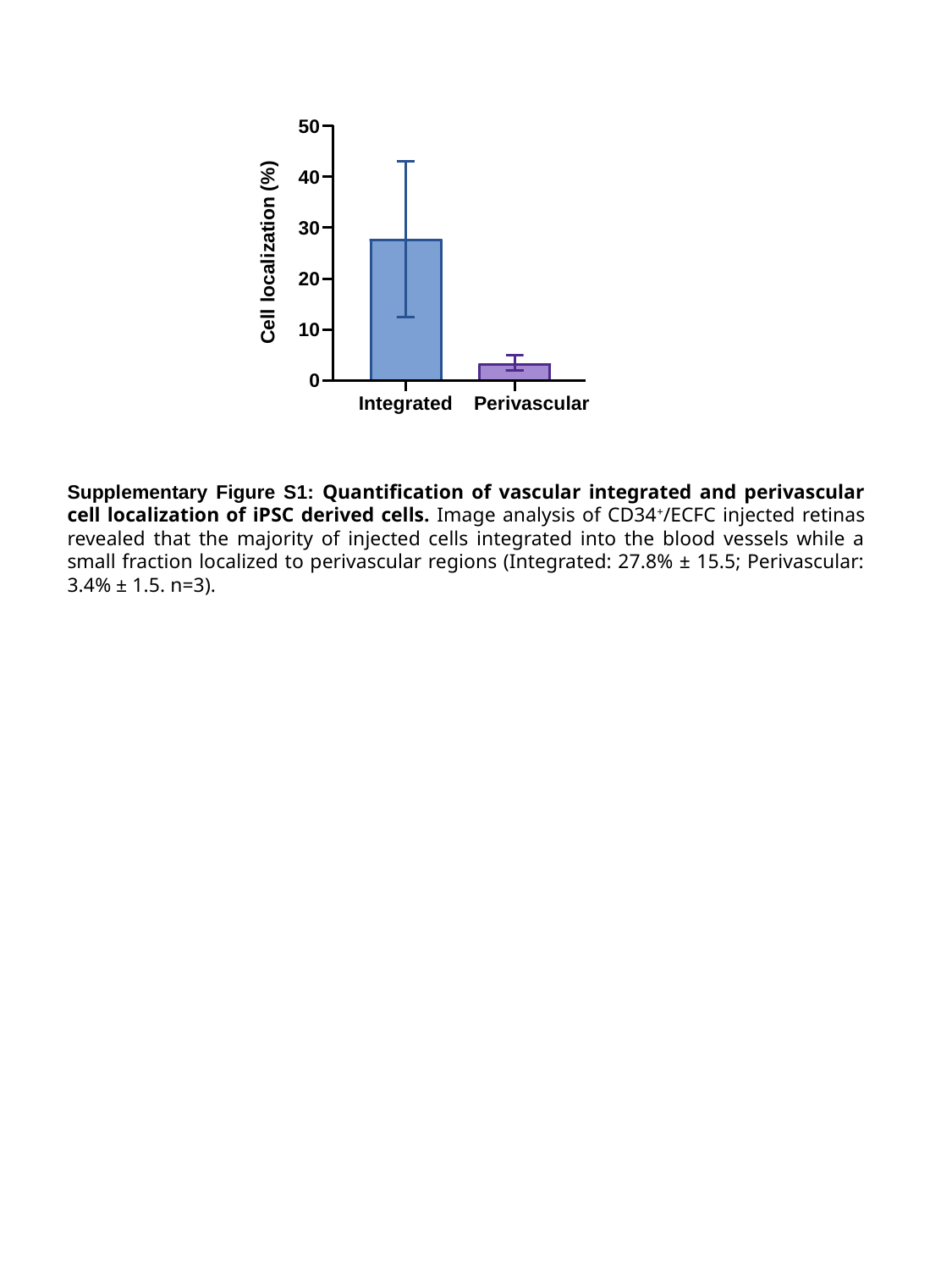

Cell localization (%)
Integrated
Perivascular
Supplementary Figure S1: Quantification of vascular integrated and perivascular cell localization of iPSC derived cells. Image analysis of CD34+/ECFC injected retinas revealed that the majority of injected cells integrated into the blood vessels while a small fraction localized to perivascular regions (Integrated: 27.8% ± 15.5; Perivascular: 3.4% ± 1.5. n=3).
