# Supplementary material for: Targeting Diabetic Retinopathy with Human iPSC-Derived Vascular Reparative Cells in a Type 2 Diabetes Model
Source: Cells. 2025 Aug 30;14(17):1352. doi: 10.3390/cells14171352 (PMC12428381; doi:10.3390/cells14171352)
Supplement: Supplementary file 1 [file cells-14-01352-s001.zip › Supplementary Table S1 _ Li Calzi et al-08-29-2025.pdf]

Supplementary Table S1: Proteomic Analysis in iPSC-Injected db/db Retinas

GeneName	feature	AntigenID	coef	P	adjustP
PDPK1	PDK1-R-V	AGID00056	0.109578958	0.029950277	0.010904848
CD274	PD-L1-R-C	AGID00300	0.089693001	0.064319002	0.019610569
MITF	MITF-R-V	AGID00403	0.066178711	0.000899953	0.000597348
CTNNB1	b-Catenin-R-V	AGID00011	0.060491549	0.000790142	0.000543707
FN1	Fibronectin-R-V	AGID00031	0.060225604	8.97E-05	9.90E-05
GJA1	Connexin-43-R-C	AGID00267	0.045728334	0.020650975	0.008152165
MMP14	MMP14-R-V	AGID00402	0.040023297	7.32E-08	7.85E-07
LYN	Lyn-R-V	AGID00439	0.033886684	7.78E-05	8.98E-05
PDCD4	Pdcd4-R-C	AGID00094	0.027531479	0.145370199	0.038753491
DDR1	DDR1-R-V	AGID00427	0.023822934	5.04E-06	1.15E-05
CREB1	CREB_pS133-R-C	AGID00393	0.022624438	0.02197646	0.008540562
GYS1	Gys-R-V	AGID00129	0.021538718	0.023931431	0.009204921
POLG	DNA_POLG-R-V	AGID00407	0.020151407	0.014402684	0.006137857
MAPK7	Erk5-R-V	AGID00372	0.020131051	0.025734896	0.00960312
MAPK1/MAPK3	MAPK_pT202_Y204-R-C	AGID02154	0.019268738	0.011468954	0.00512036
ACTB	b-Actin-R-C	AGID02143	0.017734079	1.34E-06	4.35E-06
ATP5PD	ATP5H-R-V	AGID02145	0.016720631	0.001558393	0.000913173
BAX	Bax-R-V	AGID00010	0.016686347	0.000109746	0.000114325
PAK4	PAK4-R-V	AGID02199	0.015896196	0.020823126	0.008177086
GRB7	GRB7-R-V	AGID00368	0.015621144	0.001551279	0.000913173
SGK3	SGK3-R-V	AGID00430	0.015110235	1.53E-06	4.48E-06
FABP5	FABP5-R-C	AGID02205	0.014700481	0.000402242	0.000317578
CAV1	Caveolin-1-R-V	AGID00016	0.01461547	0.136125704	0.037127307
GSK3A/GSK3B	GSK-3a-b_pS21_S9-R-V	AGID00034	0.014579879	0.005631932	0.002779072
PXN	Paxillin-R-C	AGID00054	0.014405823	0.003082132	0.001651236
MFN2	Mitofusin-2-R-V	AGID00322	0.013354845	0.149945892	0.039600643
ESR1	ER-a-R-V	AGID00335	0.013316965	0.000184568	0.000169044
HSPA9	Grp75-R-C	AGID00396	0.013181329	2.98E-05	4.00E-05
MAPK11/12/14	p38-MAPK-R-V	AGID00049	0.013062141	0.03435233	0.012069944
FOLR1	Folate-Binding-Protein-R-V	AGID02262	0.012542246	9.74E-05	0.000103185
CD44	CD44-R-C	AGID00411	0.01147535	0.116595262	0.03249322
IGF2BP3	IMP3-R-C	AGID02221	0.011368438	0.082243724	0.024478693
EPHA2	EphA2_pS897-R-C	AGID00552	0.011137499	0.019201587	0.007620112
RPS6KA1	RSK1-R-V	AGID00554	0.010810709	0.004312591	0.002200427
CSK	CSK-R-C	AGID00365	0.010710304	0.000879255	0.00058882
PHGDH	PHGDH-R-C	AGID00281	0.010514601	0.032014855	0.011544476
MAPK7	BMK1-Erk5_pT218_Y220-R-V	AGID00373	0.010066542	0.014988968	0.006280652
MYH9	Myosin-IIa-R-C	AGID00291	0.009884523	0.057341497	0.017729889
LCK	Lck-R-V	AGID00042	0.009449101	0.12412722	0.033978364
RBBP8	CtIP-R-V	AGID00367	0.009441665	0.045416573	0.014746481
PLCG1	PLC-gamma1_pS1248-R-V	AGID00516	0.009440319	0.001082747	0.000694108
TSC1	TSC1-R-V	AGID00155	0.008144196	0.088799455	0.026221805
STMN1	Stathmin-1-R-V	AGID00077	0.007575861	0.001135863	0.000704087
GLI3	Gli3-R-V	AGID00263	0.007566564	0.029686858	0.010861665
NOTCH1	Notch1-R-V	AGID02155	0.007400615	0.142614068	0.038202373
PLCG1	PLC-gamma1-R-V	AGID00512	0.006924617	0.024332783	0.009263426
GRB2	GRB2-R-V	AGID00518	0.006903501	0.000766583	0.00053238

AKT2	Akt2-R-V	AGID00316	0.006898655	0.012617355	0.005534243
TUFM	TUFM-R-V	AGID00417	0.006590685	0.174769131	0.045357895
GYS1	Gys_pS641-R-V	AGID00130	0.006560648	0.035349186	0.012179951
MYH11	MYH11-R-C	AGID00401	0.006350205	0.089584579	0.026246977
FANCD2	FANCD2-R-V	AGID00522	0.006323062	0.004899266	0.002466214
BAK1	Bak-R-C	AGID00009	0.005961069	0.103599722	0.029433416
PIK3CA	PI3K-p110-a-R-C	AGID00093	0.005789221	0.03009875	0.010905965
SMAD3	Smad3-R-V	AGID00091	0.00568796	0.018143214	0.007238396
HLA-DRA	HLA-DR-DP-DQ-DX-R-C	AGID00415	0.005662518	0.036698474	0.012398839
PRMT5	PRMT5-R-V	AGID02240	0.005465535	0.000330771	0.000272629
COL6A1	Collagen-VI-R-V	AGID00021	0.005202621	0.012830124	0.005594565
JUN	c-Jun_pS73-R-V	AGID00434	0.004757521	0.142188964	0.038202373
MTOR	mTOR_pS2448-R-C	AGID00047	0.00428896	0.183845722	0.047223338
GLUD1	Glutamate-D1-2-R-V	AGID00387	0.004046171	0.177102279	0.045804923
WEE1	Wee1_pS642-R-C	AGID00354	0.003988455	0.044171265	0.014404494
AKT1	Akt1-R-V	AGID00348	0.003881453	0.016653654	0.006751866
TFRC	TFRC-R-V	AGID00165	0.003720357	0.120467761	0.033219106
PTGS2	Cox2-R-V	AGID00195	0.003673836	0.160910687	0.042199262
YES1	YES1-R-V	AGID02183	0.00334663	0.034498459	0.012069944
FGFR1	FGFR1-R-V	AGID02249	0.002720764	0.097200034	0.028148331
TEAD1-4	TEAD-R-C	AGID00570	0.00196692	0.116968929	0.03249322
CIITA	CIITA-R-C	AGID00350	0.001781532	0.152299142	0.040081005
NOTCH3	Notch3-R-C	AGID00087	-0.00104574	0.060339072	0.018547902
TNFRSF4	CD134-R-V	AGID00358	-0.0023001	0.001994019	0.001136226
DAPK2	DAPK2-R-C	AGID00304	-0.0025117	0.022575504	0.008728141
PDK1	PDHK1-R-C	AGID00280	-0.00264675	0.14260996	0.038202373
RAD51	Rad51-R-C	AGID00360	-0.00292688	0.000863235	0.0005833
MET	c-Met_pY1234_Y1235-R-V	AGID00079	-0.00294745	0.116339909	0.03249322
SMAD4	Smad4-R-V	AGID02160	-0.00304213	1.00E-05	1.93E-05
MRAP	MRAP-R-C	AGID00273	-0.00314135	0.037932037	0.012588782
CASP3	Caspase-3-cleaved-R-C	AGID00121	-0.00318464	0.016074277	0.006660991
CDH6	Cadherin-6-R-C	AGID02261	-0.0031891	0.066863417	0.020303814
WIPI1	WIPI1-R-C	AGID00285	-0.00322199	0.123353268	0.033890191
VTCN1	B7-H4-R-C	AGID00302	-0.00332123	0.07808644	0.023427267
CRABP1	CRABP1-R-C	AGID00567	-0.00334947	0.140901869	0.038152499
HLA-DQA1	HLA-DQA1-R-V	AGID00332	-0.00354691	0.048721143	0.015616641
CDH3	P-Cadherin-R-C	AGID00055	-0.00411358	0.002214746	0.001248988
PRMT1	PRMT1-R-V	AGID01595	-0.00432629	0.069203876	0.020929784
BABAM1	MERIT40_pS29-R-V	AGID00338	-0.00433264	0.025471286	0.009552276
PTCH1	Patched-R-C	AGID00274	-0.00444673	0.000495546	0.000368001
PRKAA2	AMPK-a2_pS345-R-V	AGID00236	-0.00445512	0.036885084	0.012406004
ACLY	ACLY_pS455-R-V	AGID02114	-0.00458474	0.147258432	0.039028309
NAPSA	NAPSIN-A-R-C	AGID00216	-0.00467949	0.000654293	0.000467379
IGF1R/INSR	IGF1R_pY1135_Y1136-R-V	AGID00197	-0.00472463	0.034598536	0.012069944
PTPN11	SHP-2_pY542-R-C	AGID00183	-0.00474428	0.111303409	0.031266784
HNRNPK	HNRNPK-R-V	AGID02161	-0.00475955	0.035560365	0.012179951
IRF1	IRF-1-R-V	AGID00223	-0.00493876	0.169318919	0.04409598
FOXO3	FoxO3a_pS318_S321-R-C	AGID02197	-0.00501946	0.021281009	0.008313368
CDK9	CDK9-R-V	AGID00557	-0.00517085	0.055761366	0.01757286
DUSP4	DUSP4-R-V	AGID00253	-0.00519361	0.024454052	0.009263426
TP53	p53-R-C	AGID00050	-0.00568809	0.033215731	0.011807212

EIF2AK3	PERK-R-V	AGID00440	-0.00574538	0.099455347	0.028690677
RICTOR	Rictor_pT1135-R-V	AGID00160	-0.00582539	0.017844577	0.007157324
E2F1	E2F1-R-V	AGID00211	-0.00594621	0.057193521	0.017729889
DDR1	DDR1_pY513-R-C	AGID00428	-0.00594914	0.005919469	0.002901866
PEA15	PEA-15_pS116-R-V	AGID00125	-0.00604725	0.00916428	0.004242964
MAPK8	JNK_pT183_Y185-R-C	AGID00758	-0.00625686	0.037278213	0.012482256
MDM2	MDM2_pS166-R-V	AGID02231	-0.00627519	0.056224909	0.017588375
CDC6	Cdc6-R-V	AGID00422	-0.00627869	0.120139909	0.033219106
UVRAG	UVRAG-R-C	AGID00313	-0.00628681	0.002372366	0.001314569
FOXO3	FOXO3-R-V	AGID00378	-0.00640511	2.42E-07	1.40E-06
PARK7	DJ1-R-V	AGID00104	-0.00641691	0.100850019	0.028870925
CTNNB1	b-Catenin_pT41_S45-R-V	AGID00179	-0.00645811	0.013229161	0.005702549
MAP2K2	MEK2-R-V	AGID00201	-0.00675164	0.001681041	0.000977405
TACSTD2	TROP2-R-V	AGID02258	-0.00680399	0.028647718	0.010532849
NOTCH1	Notch1-cleaved-R-V	AGID00340	-0.00686821	0.00015222	0.000150226
CHD1L	CHD1L-R-V	AGID00447	-0.00703637	0.035563431	0.012179951
PDPK1	PDK1_pS241-R-V	AGID00057	-0.00709688	0.00899512	0.004190512
PAK4-6	PAK_pS474_S602_S560-R-V	AGID00551	-0.00719395	0.000154715	0.000150705
PREX1	PREX1-R-V	AGID00190	-0.00742038	0.055246423	0.017558126
INSR	IR-b-R-C	AGID00272	-0.00748856	0.13728086	0.037306707
STAT5A	Stat5a-R-V	AGID00069	-0.00750993	0.089575543	0.026246977
EIF4E	eIF4E_pS209-R-V	AGID00328	-0.0076165	0.000467808	0.000358799
BID	Bid-R-C	AGID00013	-0.00767774	6.21E-05	7.39E-05
PMS2	PMS2-R-V	AGID00203	-0.0077643	0.100177837	0.028788375
ATG4B	Atg4B-R-C	AGID00309	-0.00779112	0.012453794	0.005494634
SRC	Src_pY527-R-V	AGID00068	-0.00781568	0.024241674	0.009263426
RAF1	C-Raf_pS338-R-V	AGID00022	-0.00784753	0.055484616	0.017559423
SOD2	SOD2-R-V	AGID00380	-0.00798807	0.081979189	0.024478693
PTK2	FAK-R-C	AGID00030	-0.00801471	0.003970958	0.002068325
ATG3	Atg3-R-V	AGID00276	-0.0080189	0.006138929	0.002970619
PKMYT1	Myt1-R-C	AGID00318	-0.0080412	0.041131384	0.013590438
ATM	ATM_pS1981-R-V	AGID02142	-0.00814705	0.043577738	0.014272998
PRKAR1A	PKA-a-R-V	AGID00296	-0.00820513	0.093677218	0.027339267
PIP4K2A	PIP4K2A-R-V	AGID02225	-0.00842756	0.000148928	0.000148937
ERCC1	ERCC1-R-C	AGID00240	-0.0084514	0.006973404	0.003289529
HIF1A	Hif-1-alpha-R-C	AGID00419	-0.00887312	0.001131874	0.000704087
VHL	VHL-R-C	AGID00421	-0.00889736	0.034840744	0.01209817
GAB2	Gab2-R-V	AGID02272	-0.00891518	0.000182758	0.000169044
ACSS2	AceCS1-R-V	AGID00408	-0.00930467	0.033081023	0.011807212
MTOR	mTOR-R-V	AGID00046	-0.0094224	0.027281561	0.01012987
RICTOR	Rictor-R-C	AGID00159	-0.00950652	0.000911163	0.000598619
AKT1S1	PRAS40_pT246-R-V	AGID00082	-0.00960799	0.167396666	0.043747265
SLC16A3	MCT4-R-V	AGID00288	-0.00968046	0.042294432	0.013913435
AKT1	Akt1_pS473-R-V	AGID00346	-0.00968971	0.033636605	0.01190042
COG3	COG3-R-V	AGID00292	-0.0097319	1.35E-07	9.78E-07
GLS	Glutaminase-R-C	AGID00262	-0.01001772	0.002978016	0.001606935
PHLPP1	PHLPP-R-V	AGID00425	-0.01016555	0.006895908	0.003278017
BCL2A1	BCL2A1-R-V	AGID00219	-0.01033041	0.056279593	0.017588375
MSH6	MSH6-R-C	AGID00138	-0.01035515	0.025288159	0.009531256
AURKA	Aurora-A-R-C	AGID00391	-0.01038302	0.050300955	0.016054411
METTL3	METTL3-R-V	AGID02212	-0.01043929	1.50E-05	2.56E-05

SRC	Src-R-V	AGID00901	-0.01059875	0.109376263	0.030840929
LAD1	LAD1-R-V	AGID00375	-0.01064068	0.145704824	0.038753491
SIRPA	SIRP-alpha-R-V	AGID02208	-0.01070276	0.075946429	0.022876733
PRC1	PRC1_pT481-R-C	AGID00424	-0.01081398	0.016340246	0.006697204
MAP2K1	MEK1-R-V	AGID00044	-0.01085249	0.047052402	0.015211772
CDC42/RAC1	Cdc42-R-C	AGID00227	-0.0108767	0.000351679	0.000283629
AURKB	Aurora-B-R-V	AGID02195	-0.01093176	0.0964101	0.028027788
SMAD1	Smad1-R-V	AGID00113	-0.01147923	0.013623245	0.005838866
HUWE1	Lasu1-R-V	AGID00290	-0.01162675	0.010066793	0.004521272
AKT1/2/3	Akt-R-V	AGID00146	-0.01164943	0.001202721	0.000733408
ETS1	Ets-1-R-V	AGID00187	-0.01209014	0.003134293	0.001667272
ELK1	Elk1_pS383-R-C	AGID00027	-0.01218446	0.057441569	0.017729889
GCLC	GCLC-R-C	AGID00289	-0.01224626	0.0145167	0.006151494
DYRK1B	DYRK1B-R-C	AGID00571	-0.01225641	0.000143828	0.00014578
NFE2L2	NRF2-R-C	AGID00257	-0.01239551	3.43E-05	4.44E-05
NR2F2	Coup-TFII-R-C	AGID00269	-0.01242557	0.000302563	0.000254983
KIT	c-Kit-R-V	AGID02147	-0.01247635	0.086065673	0.025514993
CGAS	cGAS-R-V	AGID02101	-0.01249537	1.77E-05	2.76E-05
ARID1A	ARID1A-R-C	AGID00260	-0.01249545	3.00E-09	2.25E-07
RPA2	RPA32_pS4_S8-R-C	AGID00246	-0.01261032	3.19E-05	4.20E-05
EIF4G1	eIF4G-R-C	AGID00154	-0.01262355	0.000478483	0.000362508
HSPD1	HSP60-R-V	AGID00320	-0.01293709	0.000696489	0.000492827
AMBRA1	Ambra1_pS52-R-C	AGID00287	-0.01298836	1.65E-05	2.69E-05
PTK2B	Pyk2_pY402-R-C	AGID00275	-0.01299144	9.10E-05	9.90E-05
CDKN1B	p27_pT198-R-V	AGID00099	-0.01314747	3.51E-05	4.46E-05
EIF4E	eIF4E-R-V	AGID00078	-0.01317739	0.000603615	0.000435324
H3C1	Histone-H3_pS10-R-V	AGID00398	-0.01319315	0.032897638	0.011806045
ACACA-B	ACC_pS79-R-V	AGID00004	-0.01321996	0.015586603	0.006494788
WIPI2	WIPI2-R-C	AGID00286	-0.01324735	0.000759541	0.00053238
MACC1	MACC1-R-V	AGID00562	-0.01334288	0.006545973	0.003147282
BECN1	Beclin-R-C	AGID00377	-0.01334858	0.009575595	0.0044062
CA9	CA9-R-C	AGID00345	-0.01361371	9.07E-08	8.51E-07
TRIM28	KAP1-R-V	AGID00307	-0.01371193	2.72E-06	6.79E-06
SGK1	SGK1-R-V	AGID00431	-0.01373211	0.005374303	0.002687305
IRS1	IRS1-R-V	AGID00092	-0.01382703	0.03605054	0.012290657
RAD50	Rad50-R-V	AGID00261	-0.01413357	0.005995814	0.002920206
H2BC3	U-Histone-H2B-R-C	AGID00295	-0.01413692	0.101776901	0.029025485
MKNK1	Mnk1-R-V	AGID00122	-0.01469741	0.005422787	0.002693591
BRD4	BRD4-R-V	AGID00266	-0.01484511	0.008224215	0.003855321
AXL	Axl-R-V	AGID00215	-0.01489666	0.000180212	0.000169044
EPHA2	EphA2-R-V	AGID00438	-0.01494449	0.01004585	0.004521272
HK1	Hexokinase-I-R-C	AGID00397	-0.01502031	0.002612443	0.001420675
CD276	B7-H3-R-C	AGID00301	-0.01516992	2.70E-05	3.75E-05
PARG	PARG-R-C	AGID00374	-0.01517673	0.009705748	0.004438857
ULK1	ULK1_pS757-R-C	AGID00284	-0.0152562	0.000241036	0.000207802
EEF2K	eEF2K-R-V	AGID00136	-0.01531761	0.016182757	0.006669098
RAD17	Rad17_pS645-R-V	AGID00521	-0.01545416	2.47E-05	3.49E-05
PRKCA/B/D/E/H/Q	PKC-b-II_pS660-R-V	AGID00163	-0.01551211	0.180954146	0.046640323
CLDN7	Claudin-7-R-V	AGID00400	-0.01612603	1.74E-05	2.76E-05
MSH2	MSH2-R-C	AGID00390	-0.0163013	8.13E-05	9.24E-05
DVL3	Dvl3-R-V	AGID00116	-0.01642096	0.036248067	0.012302081

HSPA1A	HSP70-R-C	AGID00037	-0.01660628	0.000312264	0.000260234
BCL2L11	Bim-R-V	AGID00180	-0.01694861	0.00141136	0.000842563
PGM1	PGM1-R-V	AGID02207	-0.01696342	2.91E-05	3.97E-05
YWHAZ	14-3-3-zeta-R-V	AGID00101	-0.01760966	0.000288157	0.000245602
GSK3B	GSK-3B-R-C	AGID00174	-0.01774811	9.07E-05	9.90E-05
FOXM1	FOXM1-R-V	AGID02152	-0.01781859	0.001340078	0.000810577
PTEN	PTEN-R-V	AGID00061	-0.01806714	0.000184811	0.000169044
YTHDF2	YTHDF2-R-V	AGID02217	-0.01812992	5.33E-08	6.66E-07
CDK1	cdc2_pY15-R-C	AGID00314	-0.01816436	0.000228659	0.000199423
RPS6KA1	p90RSK_pT573-R-C	AGID00182	-0.01929291	0.02854376	0.010532849
CRABP2	CRABP2-R-V	AGID00568	-0.019347	1.38E-05	2.47E-05
ENO1	Enolase-1-R-V	AGID00394	-0.01935754	0.061329245	0.018775328
IGF1R	IGFRb-R-C	AGID00039	-0.01937826	0.104660671	0.029622632
SFRP1	SFRP1-R-V	AGID02179	-0.01951965	3.25E-08	6.66E-07
TP53BP1	53BP1-R-V	AGID00120	-0.01957461	0.014804319	0.00623813
IRS2	IRS2-R-C	AGID00040	-0.01960037	0.012160375	0.005396924
BIRC3	c-IAP2-R-C	AGID00278	-0.02026251	1.34E-05	2.46E-05
ATR	ATR_pS428-R-C	AGID00315	-0.02107122	5.74E-05	6.95E-05
IGFBP2	IGFBP2-R-V	AGID00038	-0.02107698	1.55E-06	4.48E-06
RPS6KB1	p70-S6K_pT389-R-V	AGID00052	-0.02124947	0.001102182	0.000700579
SRC	Src_pY416-R-V	AGID02157	-0.02195231	0.01654149	0.006742839
FLCN	Folliculin-R-V	AGID02230	-0.02217011	9.82E-06	1.93E-05
CDC25C	cdc25C-R-V	AGID00329	-0.02250845	0.000468804	0.000358799
WEE1	Wee1-R-C	AGID00317	-0.02287117	0.017718431	0.007144936
ATG5	Atg5-R-C	AGID00310	-0.02324942	0.001129707	0.000704087
TRIP13	TRIP13-R-V	AGID00426	-0.02325318	0.003591092	0.001896812
RPA2	RPA32-R-V	AGID00416	-0.02360147	0.004524882	0.002293145
CASP7	Caspase-7-cleaved-R-C	AGID00015	-0.02361738	0.002613893	0.001420675
SOX2	Sox2-R-V	AGID00298	-0.02400823	0.000197375	0.000176238
KAT2A	GCN5L2-R-V	AGID00212	-0.02447964	0.000348397	0.000283629
TSC2	Tuberin_pT1462-R-V	AGID02194	-0.02448516	0.000490774	0.000368001
CHEK1	Chk1_pS296-R-V	AGID00234	-0.0245451	3.23E-07	1.62E-06
PLK1	PLK1-R-C	AGID00084	-0.02456547	0.009907151	0.004503507
CCNB1	Cyclin-B1-R-V	AGID00024	-0.02513662	0.006905296	0.003278017
MIF	MIF-R-C	AGID00225	-0.02553993	0.004080726	0.00211084
RAB11FIP1	Rab11FIP1-R-V	AGID02232	-0.02557744	2.56E-06	6.63E-06
MSI2	MSI2-R-C	AGID00299	-0.02587275	1.08E-07	9.01E-07
WWTR1	TAZ-R-V	AGID00327	-0.02602924	0.001181743	0.000726522
MAP1LC3A/B	LC3A-B-R-C	AGID00279	-0.0262328	0.002383616	0.001314569
ACACA-B	ACC1-R-C	AGID00005	-0.02733449	1.27E-06	4.35E-06
MLKL	MLKL-R-V	AGID00312	-0.02851379	0.00091783	0.000598619
RPS6KB1	p70-S6K1-R-V	AGID00051	-0.0290127	6.26E-09	2.35E-07
MCL1	Mcl-1-R-V	AGID00198	-0.02913932	0.000832752	0.000567818
YAP1	YAP-R-C	AGID00088	-0.02917127	0.002266257	0.0012685
EIF4EBP1	4E-BP1_pS65-R-V	AGID00002	-0.02923334	0.00094259	0.000609468
RBM15	RBM15-R-V	AGID00164	-0.0296664	5.98E-06	1.28E-05
NR3C1	Glucocorticoid-Receptor-R-V	AGID00548	-0.03028203	7.29E-05	8.54E-05
PIK3R1	PI3K-p85-R-C	AGID00058	-0.03056642	2.19E-05	3.29E-05
UBAC1	UBAC1-R-V	AGID00214	-0.03083881	0.000123275	0.00012666
PARP1	PARP-R-V	AGID00370	-0.0309808	0.003658961	0.001919145
RELA	NF-kB-p65_pS536-R-C	AGID00048	-0.03119645	4.42E-05	5.44E-05

RIPK1	RIP-R-C	AGID00282	-0.03168833	2.20E-05	3.29E-05
TUBA4A	D-a-Tubulin-R-V	AGID00247	-0.03190405	0.00419755	0.002156398
EIF4EBP1	4E-BP1_pT37_T46-R-V	AGID00003	-0.03234524	6.80E-06	1.42E-05
ASNS	ASNS-R-V	AGID00264	-0.03246116	0.001415426	0.000842563
MAPK11/12/13/14	p38-MAPK-_pT180_Y182-R-V	AGID00098	-0.0343372	0.001822275	0.001051373
TSC2	Tuberin-R-V	AGID00071	-0.03445447	4.98E-08	6.66E-07
BCL2	Bcl2-R-C	AGID00433	-0.03458462	0.037856609	0.012588782
EZH2	Ezh2-R-V	AGID02273	-0.03468864	2.32E-05	3.42E-05
RRM2	RRM2-R-C	AGID00344	-0.03508397	4.18E-05	5.22E-05
ERBB3	HER3-R-V	AGID00110	-0.03517809	0.001999644	0.001136226
RB1	Rb_pS807_S811-R-V	AGID00060	-0.03538113	9.77E-05	0.000103185
YTHDF3	YTHDF3-R-C	AGID02210	-0.03661137	1.22E-06	4.35E-06
RRM1	RRM1-R-C	AGID00341	-0.03661918	0.047544235	0.01530481
EIF4EBP1	4E-BP1-R-V	AGID00001	-0.03713562	0.000187128	0.000169101
BABAM1	MERIT40-R-C	AGID00337	-0.03776639	0.000515563	0.000379112
RAD23A	Rad23A-R-C	AGID00342	-0.03784544	2.55E-06	6.63E-06
PAICS	PAICS-R-C	AGID00224	-0.03916566	0.000449265	0.000351008
LDHA	LDHA-R-C	AGID00119	-0.03948673	0.000384482	0.000306785
AKT2	Akt2_pS474-R-C	AGID00347	-0.0400502	0.012904061	0.005594565
MFN1	Mitofusin-1-R-V	AGID00321	-0.04181436	0.000175069	0.000168346
H2AX	H2AX_pS139-R-C	AGID01354	-0.04249081	1.21E-06	4.35E-06
HSPB1	HSP27_pS82-R-V	AGID00036	-0.04293953	4.78E-07	2.24E-06
PRKCA-B	PKC-a-b-II_pT638_T641-R-V	AGID00259	-0.04422333	3.16E-06	7.65E-06
MYC	c-Myc-R-C	AGID00167	-0.04469114	0.000227526	0.000199423
PDCD1	PD-1-R-V	AGID00413	-0.04477809	1.50E-05	2.56E-05
RAF1	C-Raf-R-C	AGID00188	-0.04599164	3.23E-07	1.62E-06
ATM	ATM-R-V	AGID00242	-0.04835617	5.61E-07	2.48E-06
FTO	FTO-R-V	AGID02213	-0.04865207	0.000579604	0.000422066
ATRX	ATRX-R-C	AGID00268	-0.05254239	1.43E-07	9.78E-07
MYH9	Myosin-IIa_pS1943-R-V	AGID00173	-0.05504684	4.78E-08	6.66E-07
DNMT1	DNMT1-R-V	AGID00399	-0.05863587	9.77E-06	1.93E-05
PKM	PKM2-R-C	AGID00126	-0.06293903	9.49E-07	3.96E-06
SLC1A5	SLC1A5-R-C	AGID00222	-0.06954545	1.42E-06	4.45E-06
PRKCA	PKCa-R-V	AGID00172	-0.07256578	1.20E-05	2.26E-05
WTAP	WTAP-R-V	AGID02214	-0.0747618	4.72E-06	1.11E-05
NA	PAR-R-C	AGID00245	-0.07758915	2.33E-06	6.48E-06
HK2	Hexokinase-II-R-V	AGID00442	-0.09389037	1.64E-05	2.69E-05
SYP	Synaptophysin-R-C	AGID00565	-0.116804	2.41E-05	3.48E-05
ALKBH5	ALKBH5-R-V	AGID02215	-0.13471405	1.66E-07	1.04E-06
ARHGAP45	HMHA1-R-V	AGID00333	-0.21116355	5.72E-06	1.26E-05
VASP	VASP-R-V	AGID00073	-0.27226934	1.32E-06	4.35E-06
